# Cardiovascular Mortality in 10 Cohorts of Middle-Aged Men Followed-Up 60 Years until Extinction: The Seven Countries Study

**DOI:** 10.3390/jcdd10050201

**Published:** 2023-05-02

**Authors:** Alessandro Menotti, Paolo Emilio Puddu, Anthony G. Kafatos, Hanna Tolonen, Hisashi Adachi, David R. Jacobs

**Affiliations:** 1 Association for Cardiac Research, 00182 Rome, Italy; amenotti2@gmail.com; 2 EA 4650, Signalisation, Electrophysiologie et Imagerie des Lésions d’ischémie Reperfusion Myocardique, Université de Normandie, 14032 Caen, France; 3 Department of Social Medicine, Preventive Medicine and Nutrition Clinic, University of Crete, 71003 Heraklion, Greece; 4 Department of Public Health and Welfare, Finnish Institute for Health and Welfare, 00271 Helsinki, Finland; 5 Department of Internal Medicine, Division of Cardio-Vascular Medicine, Kurume University School of Medicine, Kurume 830-0011, Japan; 6 Division of Epidemiology and Community Health, School of Public Health, University of Minnesota, Minneapolis, MN 55455, USA

**Keywords:** mortality, CVD, CHD, STROKE, heart diseases of uncertain etiology, age at death, risk factors, male cohorts, 60-year follow-up

## Abstract

Objectives. To investigate mortalities from three major groups of cardiovascular diseases (CVDs) in a pooled cohort and followed up until extinction. Materials and Methods. Ten cohorts of men (*N* = 9063) initially aged 40–59, in six countries, were examined and followed-up for 60 years. The major CVD groups were coronary heart disease (CHD), cerebrovascular diseases (STROKE) and other heart diseases of uncertain etiology (HDUE). Results. Death rates from CHD were higher in countries with high serum cholesterol levels (USA, Finland and The Netherlands) and lower in countries with low cholesterol levels (Italy, Greece and Japan), but the opposite was observed for STROKE and HDUE, which became the most common CVD mortalities in all countries during the last 20 years of follow-up. Systolic blood pressure and smoking habits were, at an individual level, the common risk factors for the three groups of CVD conditions, while serum cholesterol level was the most common risk factor only for CHD. Overall, death rates for the pooled CVDs were 18% higher in North American and Northern European countries, while CHD rates were 57% higher in the same countries. Conclusions. Differences in lifelong CVD mortalities across different countries were smaller than expected due to the different rates of the three groups of CVD, and the indirect determinant of this seemed to be baseline serum cholesterol levels.

## 1. Introduction

The Seven Countries Study of Cardiovascular Diseases (SCS) was started in 1958, and the attention of the investigators was initially focused on coronary heart disease (CHD) with significant differences across countries, largely explained by classical risk factors and lifestyle habits, such as diet, smoking habits and physical activity [1,2,3,4]. Only later, and to a lesser extent, attention was given to other types of cardiovascular diseases (CVDs) [5,6,7,8,9,10]. On the one hand, overall, the different types of CVD mortality vary (both per geographic areas and in time). On the other hand, it is not frequently considered that risk factors also differ per type of CVD with the consequence that mixing up all individual outcome types may attenuate potentially relevant aspects of prediction and impact when specific interventions are performed.

Presently, 10 of the 16 original SCS cohorts of middle-aged men have reached a follow-up of 60 years with practical extinction, which has no counterpart in the current literature, and thus, prevents comparisons and reference to different investigations that only share a few elements with SCS but not long-term duration and specific outcome types when assessing mortality. In this paper, we describe and interpret the ultimate mortality status for all these men, according to selected and specific types of lifelong CVD mortality.

## 2. Materials and Methods

### 2.1. Populations and Measurements 

The analysis was conducted on 10 of the 16 cohorts of men in the SCS that reached a follow-up for life status and mortality of 60 years. The cohorts included were the U.S. railroad workers; two rural cohorts in Finland (East and West Finland); one sample of men from the commercial town of Zutphen, The Netherlands; two rural cohorts in Italy (Crevalcore and Montegiorgio); two rural cohorts in Greece (Crete and Corfu); one cohort from a rural village and one cohort from a fishing village in Japan (Tanushimaru and Ushibuka, respectively). All the men were aged 40 to 59 years at the entry examination held between 1958 and 1961 and the participation rate of those invited ranged from 75% to 100%, with an average of around 95%. More details can be found elsewhere [1,2,3].

The risk factors measured in all cohorts and used in this analysis were: (1) age, in years approximated to the nearest birthday; (2) physical activity at work, derived from a few questions combined with the reported occupation, and classified as sedentary, moderate or vigorous (in percent), and, in one country, this classification was indirectly validated by ergonometric measurements [11] and energy intake derived from dietary history [12]; (3) smoking habits derived from a standard questionnaire, and classified as never smokers, ex-smokers and current smokers (in percent); (4) body mass index, derived from height and weight measured following the procedure of the WHO’s *Cardiovascular Survey Methods* manual (WHO Manual), in kg/m^2^ [13]; (5) systolic blood pressure measured in supine position at the end of a physical examination using mercury sphygmomanometers, following the procedure described in the WHO Manual [13] (in mmHg) (the average of two measurements taken one minute apart was used for analysis); (6) heart rate, derived from a resting ECG (in beats/min); (7) serum cholesterol level measured on casual blood samples following the technique of Anderson and Keys [14]; (8) prevalence of cardiovascular diseases (CVDs) as defined by the CVD prevalence criteria of the SCS [1], expressed as 1 = yes or 0 = no; (9) prevalence of silent major ECG abnormalities in subjects without a diagnosis of CVD, including any of the following Minnesota Code items, edition 1968 [13], 1.1, 1.2, 5.1, 5.2, 6.1, 7.1, 7.4, 8.3, corresponding to major Q waves, major negative T waves, 3rd degree AV block, left ventricular branch or intraventricular block, and atrial fibrillation, and expressed as 1 = yes or 0 = no.

Life status and mortality had been checked for 60 years and, among 9063 men, only 3 men were still alive and 68 were lost to follow-up at defined dates, and censored (0.8%). As a consequence, the analysis was conducted on 99.2% of the men enrolled at baseline. Specifically, losts to follow-up were 4.3% in Japan, 0.70% in the USA and between 0.20% and 0.06% in the other countries. Therefore, some caution should be taken in the evaluation of the Japanese data.

Causes of death were allocated by reviewing death certificates, and when available, combining that basic information with the data of interim examinations, hospital and medical records, interviews with physicians and relatives of the deceased and any other witnesses of fatal events. Causes of death were determined by a single coder following defined criteria, and employing the 8th revision of the WHO-ICD (ICD-8) [15]. In the presence of multiple causes and uncertainty about the principal cause, a hierarchical preference was adopted with violence, cancers, CHD, STROKE and others, in that order.

Cardiovascular mortality end-points were chosen as follows using the 8th revision of the WHO-ICD [15]: (a) coronary heart disease (CHD) included cases of myocardial infarction, acute ischemic attacks, and sudden coronary death, after the exclusion of other possible causes (ICD-8 codes 410, 411, 412, and 795), and cases with only mention or evidence of chronic or other types of CHD (part of code 412 and code 414) were not included in this group for reasons given elsewhere [7,8,9,10], while healed myocardial infarction was retained; (b) cerebrovascular diseases (STROKE) included any type of cerebrovascular disease (ICD-8 codes 430–438); (c) heart disease of uncertain etiology (HDUE) included a pool of symptomatic heart diseases (ICD-8 code 427) (heart failure, arrhythmia, blocks, covering 64% of all cases), ill-defined hypertensive heart disease (usually in the absence of documented left ventricular hypertrophy, covering 8% of cases) (ICD-8 codes 402–404) and cases quoted as chronic or other types of coronary heart disease, without any indication of typical coronary syndromes (ICD-8 part of codes 412 and 414) (covering 28% of cases). The pool of the three selected CVD end-points covered 92% of all CVD deaths in 60 years. All other rare or etiologically defined cardiovascular diseases were not included in this analysis. For some specific analyses, we combined STROKE rates with HDUE rates to obtain a single counterpart (STHD) versus CHD.

The reason for separating CHD versus HDUE is that these two conditions differ from each other with respect to several characteristics [7,8,9,10], which include appearance and age at death, typically higher in HDUE than in CHD; serum cholesterol level predicts CHD but not HDUE; Mediterranean diet and vigorous physical activity are protective of CHD but not of HDUE.

Baseline measurements were taken before the era of the Helsinki Declaration and approval was implied by participation, while in subsequent examinations, verbal or written consent was obtained for collection of follow-up data.

### 2.2. Statistical Analysis 

Analyses were performed on the 10 cohorts or, alternatively, on data represented by the 6 countries involved, combining cohorts belonging to the same country. The second option made some findings easier to describe and interpret and was further justified by the similarity of characteristics of cohorts belonging to the same country. The framework that was used is stated for each analysis.

Approximately 4 per 1000 of baseline measurements were missing and were imputed using the multivariate normal procedure. Baseline risk factor measurements in the 6 countries were described as means or proportions for each country and on total cases and compared across cohorts by an ANOVA or chi-squared test. A sensitivity analysis was performed on the ANOVA and chi-squared test.

Death rates for the 3 CVD end-points, the combination of STROKE + HDUE (STHD) and the pool of the 3 end-points were computed for 3 partitioned time periods of 20 years each and during 60 years in all cohorts combined and separately in the 6 countries.

Weibull distribution hazard rates were computed for CHD, STROKE and HDUE mortalities in the pool of all cohorts during the 60-year follow-up.

Age at death for each CVD end-point was computed and compared across end-points and across countries, and tested by an ANOVA. The outcome was evaluated by using a sensitivity analysis.

Cox proportional hazards models were used to produce individual predictions of events with CHD, STROKE, HDUE separately as dependent variables and all risk factors plus 2 reference categories as predictive covariates. Moreover, dummy variables that identified the 6 countries were added (Finland as a reference). A test of heterogeneity (across countries) of multivariate coefficients was computed for selected risk factors. Another independent Cox model was used with non-CHD death as the end-point.

On all Cox models, the ROC curves were computed.

A discriminant analysis model was solved using CHD deaths and STHD deaths as end-points and the usual risk factors as predictors, pooling all the 10 cohorts together. This analysis is a multiple linear regression with 2 categorical dependent variables and a common set of independent variables that automatically compare the 2 end-points. This method was a way to challenge 2 large CVD groups versus CHD, taking into account the different roles of serum cholesterol level, beyond other characteristics.

A simple ecological analysis based on linear correlation was performed using death rates from CHD, STROKE and HDUE in the 10 cohorts versus the mean levels of the 3 major risk factors, i.e., serum cholesterol, systolic blood pressure and prevalence of smoking habits.

The mean entry levels of serum cholesterol for subjects who later died from CHD, STROKE or HDUE were computed and compared for each country, and an ANOVA across countries was computed and tested with a sensitivity analysis

## 3. Results

### 3.1. Baseline Risk Factors and Total Mortality

The mean risk factor levels at entry examination for the six countries are summarized in Table 1, where their large heterogeneity across countries appears, as documented by the ANOVA and chi-squared test. In general, most factors have higher levels in the USA and the Northern European countries, and lower levels in Southern Europe and Japan. However, there were exceptions, such as the prevalence of sedentary physical activity that was low in Finland or the prevalence of smokers that was high in Japan. The largest difference was observed for serum cholesterol level, where the gap in countries with extreme levels was almost 100 mg/dL. More details can be found in previous contributions [1,2,3].

The ANOVA sensitivity analysis, removing one country at a time, did not show substantial changes in the *p*-values, even in the case of extreme differences across countries, such as for serum cholesterol level, vigorous physical activity and CVD prevalence. Only for ECG abnormalities, the *p*-value was significantly changed when the Netherlands was removed.

Table 1 also lists the 60-year all-cause death rates percent, as a reference for the subsequent Table 2. Almost surely, the lower rates in Japan depend upon the large losses to follow-up.

### 3.2. CVD Death Rates Time Trends in Six Countries 

Death rates for the CVD end-points in the six countries and in their pool are presented in three partitioned independent periods of 20 years each and for a full 60 years (Table 2). Note that the men alive at the beginning of the three time periods were aged 40–59, 60–79 and 80–99 years, respectively.

**Figure 1:**
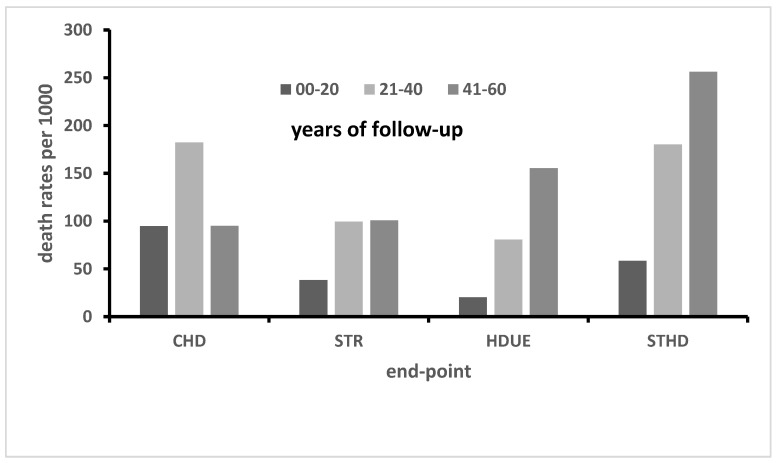
Death rates from CHD, STROKE, HDUE and STHD during 3 periods of 20 years each, in the pool of 10 cohorts.

From the pool section, it appears that the CHD death rate increased from the first to the second 20-year period, and then declined. In contrast, the death rates from STROKE and HDUE continuously increased across the three time periods. In general, this was also true for each of the six countries, with the exception of Greece, where the CHD death rate also increased from the second to the third time period. At the end-point, due to this trend, all CVD death rates in Greece became larger than in The Netherlands, Italy and Japan. The reason for this finding has been documented and explained in a previous analysis on 50 years of follow-up, since, in that country, large increases in the risk factor levels and, in particular, serum cholesterol level were recorded during the first 35 years of follow-up, corresponding to an acceleration of CHD death rates described by the shape parameter of the Weibull distribution [16]. The overall trend suggested that STROKE and HDUE largely substituted CHD as the major CVD fatal events during the late part of follow-up. A particular feature of the data indicates that the ratio of CHD/STHD was high at the beginning of the follow-up (>1) and overall in countries with high baseline serum cholesterol levels (i.e., >235 mg/dL in USA, Finland and The Netherlands), and the ratio declined to levels <1 during the follow-up. The trend was different for the three countries with low baseline serum cholesterol levels (i.e., <210 mg/dL in Italy, Greece and Japan) where the ratio of CHD/STHD was initially and overall <1, still declining subsequently (except in Greece). A graph describing this phenomenon using the pool of all cohorts is reported in Figure 1. The possibility of the phenomenon of competing risks cannot be excluded; however, the problem was not explored in the present investigation.

Another effect of the abovementioned trends was that the ratio of extreme death rates across countries was 4.0 considering only CHD, but became 1.7 when the pool of the three major CVDs was considered. In addition, the death rate for the pool of the three CVDs was only 18% greater in the high serum cholesterol countries than in the low serum cholesterol countries, while the excess was 57% for CHD death rates. Moreover, in Greece, the pool of the three major types of CVD death rates was larger than in The Netherlands, Italy and Japan.

### 3.3. Weibull Hazard Rates for CVD Mortality 

**Figure 2:**
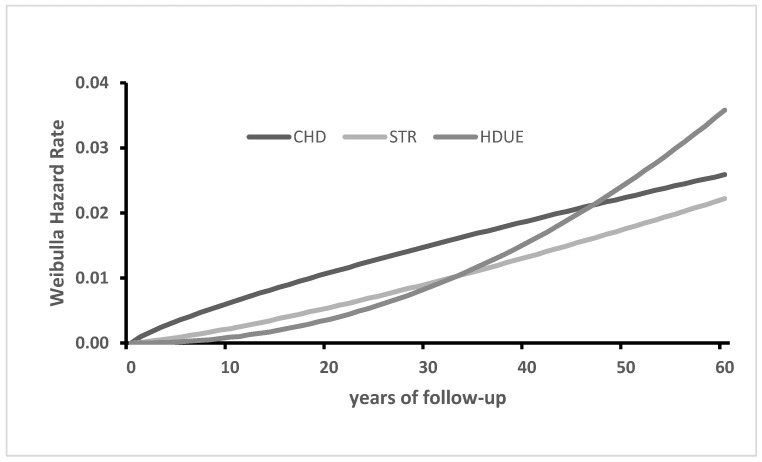
Weibull distribution hazard rates for CHD, STROKE and HDUE mortality in 60 years in the pool of 10 cohorts.

A more sophisticated way to look at these trends was considered by analyzing the data with the Weibull distribution whose hazard rates are reported for CHD, STROKE and HDUE in the pool of all cohorts for 60 years (Figure 2). Overall, the hazard rate for CHD slightly declined during the second part of the follow-up, while the reverse happened for STROKE and, especially, for HDUE whose increase was relevant. Similar trends were obtained for the single countries (details not reported), with the exception of Greece where all end-points showed increasing hazard rates, perhaps due to the documented and explained increase in risk factor levels during the first 30–35 years of follow-up [16].

### 3.4. Age at Death in Different CVDs 

Age at death computed for each end-point and for the six countries showed significant differences (Table 3). In particular, the mean age at death across the three CVD end-points was lower for CHD in all countries except in Greece, and definitely higher for STROKE and HDUE. Comparing single end-points across the six countries, age at death for CHD was lowest in Finland and highest in Greece; for STROKE, the lowest level was in Japan and the highest level was in Greece, and the same applied to HDUE. Age at death in the pool of CVDs had closer extremes (i.e., 72.9 in Finland and 80.6 in Greece). The sensitivity analysis across countries for the three end-points, removing each country one-by-one, did not show any significant variation compared with the basic findings. The same procedure across end-points had little meaning due to the presence of only three units.

### 3.5. Cox Model Predicting CVD Mortality 

Before using the Cox proportional hazard models, tests were conducted to evaluate possible distortion of coefficients due to abnormalities of baseline and follow-up data mainly related to the Japanese cohorts, producing the following comparisons: (i) coefficients of the main models (for the three CVD end-points) including dummy variables for the identification of cohorts versus coefficients of models not including those dummy variables, with only one risk factor having a significantly different coefficient (serum cholesterol level in the CHD model); (ii) coefficients of the main models (for the three CVD end-points) versus coefficients of models with exclusion of the Japanese cohorts (being those with the greater losses to follow-up), with no significant differences for any risk factor.

After that, models were computed for each CVD end-point with all risk factors and two reference categories as covariates plus dummy variables of countries (Finland as a reference) (Table 4). The significant coefficients for CHD were age, systolic blood pressure, current smokers, moderate and sedentary physical activity, CVD prevalence and silent ECG abnormalities, plus serum cholesterol level. For STROKE, only age, systolic blood pressure, current smokers and heart rate were significant, while for HDUE, systolic blood pressure, current smokers, CVD prevalence and silent ECG abnormalities were significant.

Only three risk factors had significant coefficients for each of the three end-points (age, systolic blood pressure and current smokers), while the coefficient that mostly varied across end-points was that of serum cholesterol level which was predictive only for CHD. Tests of heterogeneity of coefficients across the three end-points were performed for serum cholesterol level, systolic blood pressure and smoker prevalence, all being highly significant.

The independent Cox model with non-CHD deaths as the end-point (Table 5) showed a loss of significance for serum cholesterol level, while several other non-specific risk factors maintained their predictive role. However, only dedicated and planned analyses that deal with competing risks can solve the problem of possible competing risks.

The ROC curves for the Cox models had moderate or low levels, that is, 0.65 for CHD, 0.59 for STROKE, 0.54 for HDUE and only 0.51 for non-CHD events.

### 3.6. Discriminant Function in Different CVD Mortality End-Points 

We explored the role of serum cholesterol level in another way, that is, using the discriminant function with CHD and STHD mortality as end-points related to all risk factors. Among the various findings, Table 6 reports part of the variable influence section where the parameter *lambda* is a test that measures the impact on discrimination when removing a variable or when a single variable is the only independent variable used. The F-values test the significance of *lambda* and the F-probabilities are the correspondent probabilities of F-values. Low probabilities (for example <0.05) correspond to the importance of the variable in discriminating the two end-points. The data clearly showed that serum cholesterol level was the most important variable in discriminating the two end-points, followed by CVD prevalence and current smoker prevalence. Moreover, within the discriminant function, the regression coefficients of serum cholesterol level for the two end-points have opposite algebraic signs.

### 3.7. Ecological Analysis of Risk Factors Versus CVD Mortality 

**Figure 3:**
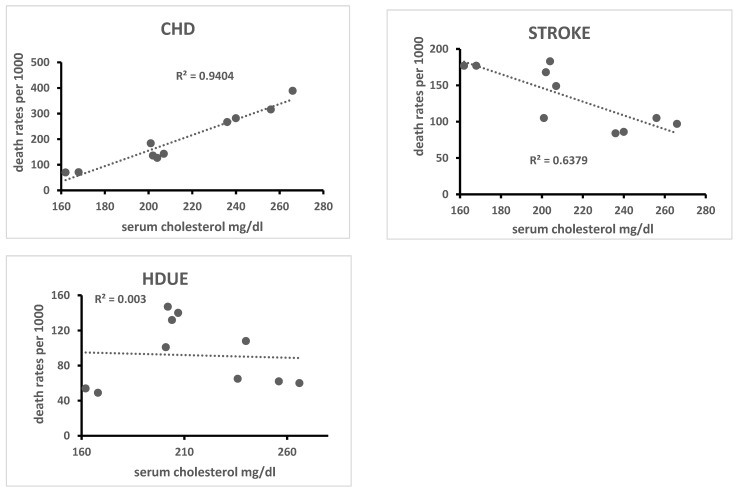
Ecological relationship of baseline serum cholesterol level with 60-year CHD, STROKE and HDUE mortality in 10 cohorts of 6 countries.

**Figure 4:**
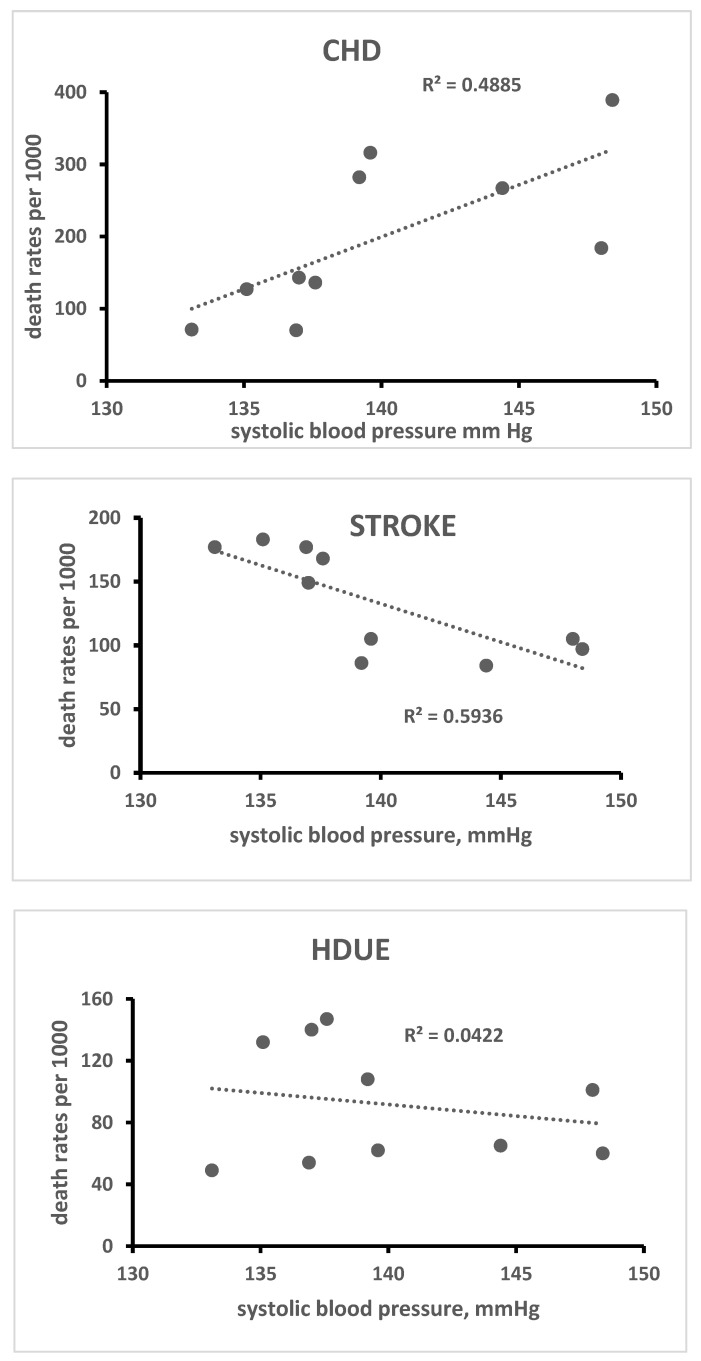
Ecological relationship of baseline systolic blood pressure with 60-year CHD, STROKE and HDUE mortality in 10 cohorts of 6 countries.

**Figure 5:**
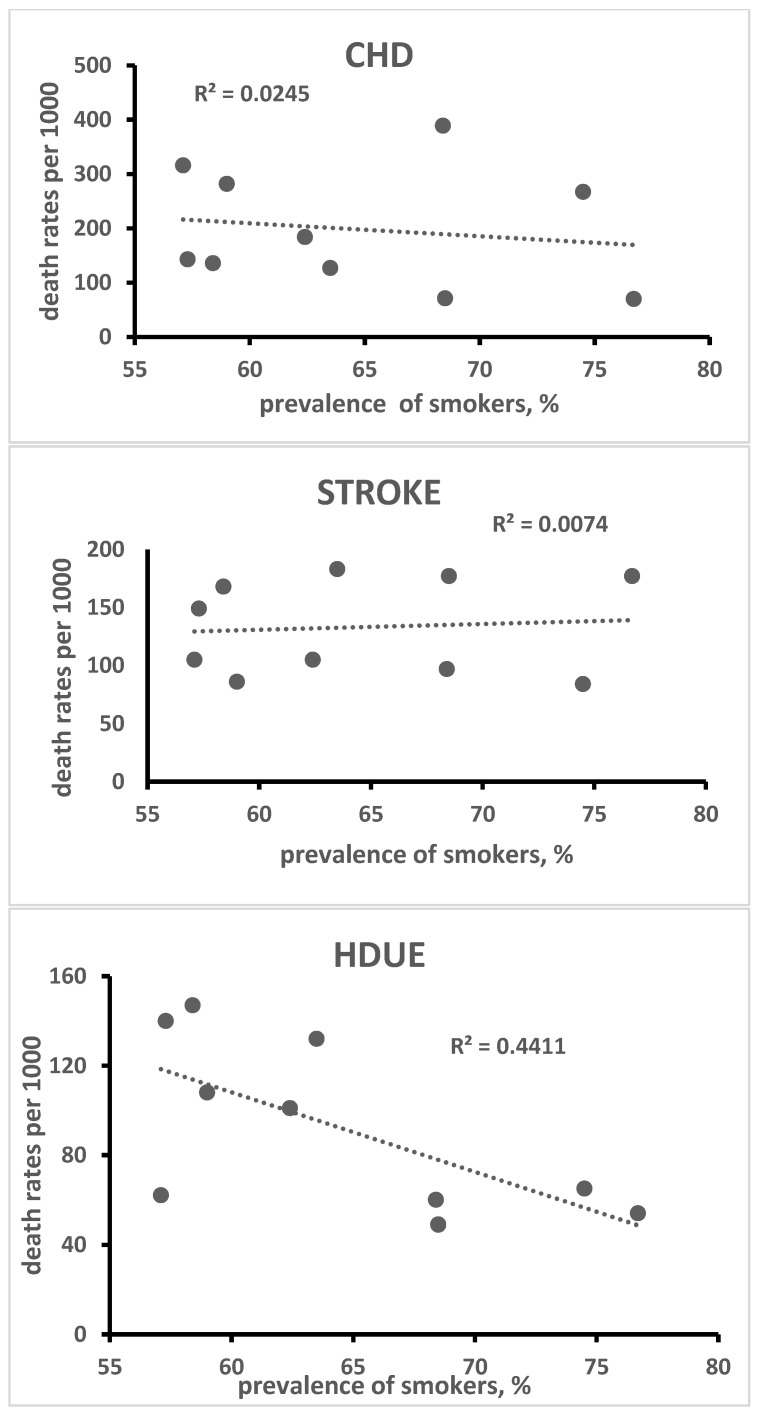
Ecological relationship of baseline prevalence of smokers with 60-year CHD, STROKE and HDUE mortality in 10 cohorts of 6 countries.

A simple ecological analysis was conducted using all 10 cohorts, some of their risk factors and CVD death rates for the three types of disease. In particular, linear correlation coefficients were computed and plotted for visual examination of serum cholesterol level, systolic blood pressure and current smoker prevalence versus 60-year death rates from CHD, STROKE and HDUE (Figure 3, Figure 4 and Figure 5).

The ecological relationship of serum cholesterol level was high, direct and significant for CHD (R = 0.98, *p* < 0.0001) and high, inverse and significant for STROKE (R = −0.80, *p*= 0.0027); systolic blood pressure was again high, direct and significant for CHD (R = 0.70, *p* = 0.0121) and high, inverse and significant for STROKE (R = −0.77, *p* = 0.0046); the prevalence of smokers was inversely and significantly associated only with HDUE. Again, from the ecological point of view, we found, across the 10 cohorts, a strong inverse and significant relationship of death rates from CHD versus those from STHD (R = −0.71, *p* = 0.0054). Using the product of systolic blood pressure times serum cholesterol level as the independent variable, the R was 0.99 for CHD, −0.84 for STROKE and −0.11 for HDUE. However, the interaction terms could not be independently tested due to the small number of statistical units. For the same reason, an evaluation using a sensitivity analysis determined unacceptable losses of degrees of freedom and of significance.

### 3.8. Baseline Serum Cholesterol Level in Men Who Later Died from Different CVDs 

Since baseline serum cholesterol level seemed to be, at least, an indirect determinant of different death rates for the three CVD end-points, another rough but clear way to show its role is given in Table 7, where the mean entry levels of serum cholesterol are reported for each CVD end-point. Clearly, in each country, average levels were higher for CHD, and definitely lower for the other two end-points.

The sensitivity analysis for ANOVA across countries did not change the situation due to the largely different entry levels of serum cholesterol.

## 4. Discussion

The initial date and the length of follow-up of this study have a historic meaning since similar investigations probably do not exist. As a consequence, nomenclature of diseases, diagnostic criteria and ICD codes were those adopted in the 1960s, and then were left unchanged in order to maintain comparability of data and findings.

In this analysis, we included all men enrolled at baseline without excluding those carrying a prevalent CVD, since the purpose was to describe the natural history of CVD mortality in whole population groups.

The selection of cohorts was made with the aim to find differences in CHD incidence and mortality and in personal characteristics, not necessarily representing the countries. Therefore, the mention of countries is simply to identify the location of cohorts. Incidentally, when more cohorts were selected per country, their characteristics were rather similar. Moreover, it is true, for example, that the sample prevalently made of farmers and another sample of fishermen do not represent the present situation of Japan. However, if we want to use the present demographic, occupational and economic situation in a country such as Japan (or elsewhere) to learn how they have evolved in the long term, another study should be conducted and monitored for the next 60 years. The SCS can, therefore, be defined as a historic study, in the hope that something may be learned from history.

Only men were enrolled in this study, since the presence of women would have required using very large numbers in the hope to obtain sufficiently large incidence-mortality data during a reasonable period of time.

Large differences in CHD death rates were seen across countries, with higher levels in North American and Northern European countries, but the opposite was found for STROKE and HDUE, a situation that could be conditioned by different levels of baseline serum cholesterol. Moreover, age at death was low for CHD mortality, high for HDUE and intermediate for STROKE.

The major risk factors common to the three end-points were systolic blood pressure and smoking habits, while serum cholesterol level was a risk factor only for CHD.

Some caution should be taken in the interpretation of the Japanese mortality data due to the large loss of men during the follow-up. The problem is evident by looking at the definitely lower all-cause death rates reported in Table 1. However, we are more confident in the multivariate Cox models and in the discriminant function where the Japanese problem is likely diluted into the larger contribution of other cohorts, as described in the Results section.

Beyond the known significant differences across countries in some CVD risk factor levels, we confirmed, on the one hand, that CHD death rates were higher in North American and North European countries. On the other hand, and this was partially a surprise, death rates from STHD and HDUE were lower in those countries and higher in South Europe and Japan. As a consequence, the pool of major CVD mortality did not differ as much as expected by the CHD component. The advantage of countries with low CHD is seen in the older age at death due to STROKE and HDUE despite their higher rates.

A peculiarity of this scenario was that countries with low CHD rates and high STROKE and HDUE rates had baseline serum cholesterol levels definitely lower than those of the countries with high CHD rates. Moreover, the ratio of CHD death rate/STHD was always lower in the countries with low baseline serum cholesterol levels than in the other countries, although a tendency to decline along time was also seen in the countries with high baseline serum cholesterol levels.

The older age at death of STROKE and HDUE versus CHD within each country suggests that men in those cohorts tended to have different pathophysiological conditions. The different time trends of those three CVD mortalities were confirmed by another approach, i.e., the estimate of hazard rate following the Weibull distribution, where that of HDUE overtook the other two components in the last part of the follow-up. This seems to make sense, since heart failure and important arrhythmias are both most typical in older people.

The special role of population and individual serum cholesterol levels on rates and prediction of the three CVD end-points was reiterated by the findings of the Cox models, where it appears that serum cholesterol level can significantly predict CHD events, while this is not the case for STROKE and HDUE. A limitation is that we were unable, in the majority of STROKE cases, to separate thrombotic from hemorrhagic events.

Altogether, few risk factors were significantly predictive in the long follow-up duration. Only blood pressure and smoking habits were both predictive of STROKE and HDUE, which means they may be considered to be more universal risk factors for any major CVD condition than serum cholesterol level. The Cox model with the non-CHD deaths as end-point simply confirmed that serum cholesterol level predicts only CHD in a probably specific way. Another indication of the specific role of serum cholesterol level came from the discriminant analysis where the test lambda suggested its primary role in discriminating CHD from the pool of STHD.

There were limitations associated with the simple ecological analysis due to the small number of statistical units. Nevertheless, it appeared clear that population levels of serum cholesterol were positively associated with CHD mortality, which was not the case for STROKE and HDUE whose relationships were negative or null. The type of association for these two end-points can be the consequence of a competing risk phenomenon, which, on the Italian data, have already been documented with specific analyses [17,18]. In particular, in a 50-year follow-up, specific Fine and Gray models suggested a critical role of serum cholesterol level, which was directly associated with CHD and inversely with other causes of death.

The data described here deserve a dedicated analysis on competing risks using proper models, that is planned and in due course with their complex procedures.

The inverse association of blood pressure with STROKE mortality is a paradox that has already been documented in this study using the 25-year follow-up data, despite a strong correlation at the individual level [6]. Again, when comparing populations instead of individuals, this finding can partly be explained by the fact that at the population level (10 cohorts), the linear correlation coefficient of systolic blood pressure with serum cholesterol level was 0.59 (*p* = 0.0363). In this case, serum cholesterol level seems to dominate the picture by capturing many cases of CHD, thus, automatically reducing the risk of STROKE and HDUE. From the ecological point of view, the prevalence of smokers did not segregate cohorts with high versus low CVD risk except for HDUE, because high prevalence of smokers was also found in the Japanese cohorts whose CVD risk was low. Nevertheless, smoking at the individual level predicted early deaths due to all CVD outcomes.

During the long follow-up, changes in risk factor levels occurred largely due to aging with variable trends for systolic blood pressure and serum cholesterol level that, over the first 35 years, were increasing and subsequently going flat or decreasing [19]. This was true for the European countries, since, for the U.S. and Japanese cohort data on risk factors, changes were available only for a maximum of 10 years, while in most European countries, cohorts data were available up to 35–40 years.

Previous analyses have described the coherent influence of risk factor changes on CHD or STROKE mortality during various periods of follow-up, mainly related to serum cholesterol level and systolic blood pressure [5,20,21,22]. In addition, analyses studying baseline risk factor levels in subsequent partitioned periods of follow-up have suggested the existence of a long-term duration of risk factors, with predictive power up to 40 years of follow-up, although slightly declining over time [23,24,25,26,27,28]. In another paper [29], data of systolic blood pressure changes were combined with the long-term prediction of baseline levels and it was concluded that the two approaches together provided a better description of the observed facts. The most comprehensive findings on this issue, although limited to nine European cohorts, were found in an analysis where the scores of major CVD risk factors changes during the first 35 years of follow-up were well correlated with the shape parameter of the Weibull distribution that described the acceleration or deceleration of hazard rates over 50 years [16]. Some of the quoted changes in risk factors influencing CVD mortality explain part of time trends of mortality described in this analysis.

The literature does not offer any contribution dealing with cohorts that have been followed long enough to reach extinction, thus, allowing a full description of life-long diseases and a proper comparison with our findings. Moreover, a comparison with our findings would also be impossible because HDUE is usually disregarded due to the common habit of pooling all CVDs together simply because they are related to the same anatomic and physiologic system [30].

## 5. Conclusions

In general, there are at least three main results from this life-time analysis of CVD mortality in extinct cohorts, originally composed of middle-aged men in different countries and cultures: (1) The differences in CHD mortality across populations are significant but, across different cohorts, opposite differences are found for STROKE and HDUE mortality. (2) Populations (and individuals) with low baseline serum cholesterol levels have a lower risk of CHD mortality but a definitely higher risk of STROKE and HDUE mortality, largely compensating for the difference in CHD mortality, but having the advantage of higher age at death for the last two CVD conditions, which also means that avoidance of CHD death may not fully protect against death due to other CVD causes. (3) The difference in overall mortality across countries for the pool of major types of CVDs is not as large as popularized, and the contributions of the three disease groups are different as a function of population serum cholesterol level. Despite this, the universal risk factors for the major types of CVDs are still systolic blood pressure, even in populations with relatively low mean levels, and smoking habits.

## Figures and Tables

**Table 1:** Baseline risk factors expressed as mean and standard deviation (continuous variables) or proportion (%) and standard error (discrete variables). The 60-year all-cause death rates are listed in the last line.

	USA	Finland	The Netherlands	Italy	Greece	Japan
*N*	2571	1677	878	1712	1215	1010
Age	49.4 (5.8)	49.4 (5.5)	49.9 (5.5)	49.1 (5.1)	49.3 (5.6)	49.8 (5.7)
Sedentary phys act, %.	66.0 (0.9)	10.2 (0.7)	24.1 (1.4)	9.7 (0.7)	17.7 (1.1)	5.7 (0.7)
Moderate phys act, %.	34.0 (0.9)	15.7 (0.9)	64.6 (1.6)	22.1 (1.0)	33.7 (1.4)	29.4 (1.4)
Vigorous phys act, %.	0	74.1 (1.1)	11.3 (1.1)	68.2 (1.1)	48.6 (1.4)	64.9 (1.5)
Never smoker, %.	19.9 (0.8)	18.8 (1.0)	7.3 (0.8)	25.4 (1.1)	24.1 (1.2)	15.2 (1.1)
Ex-smoker, %.	21.1 (0.8)	18.3 (1.0)	18.2 (1.3)	13.6 (0.8)	15.9 (1.1)	9.9 (0.9)
Current smoker, %.	59.0 (1.0)	62.6 (1.2)	74.5 (1.5)	60.7 (1.2)	60.0 (1.4)	72.6 (0.9)
Body mass index	25.2 (3.2)	23.7 (3.2)	24.0 (2.7)	25.2 (3.7)	23.1 (3.2)	22.0 (2.4)
Systolic blood pressure, mmHg	139.2 (20.8)	143.9 (20.7)	144.4 (19.8)	143.6 (21.0)	136.2 (20.5)	135.0 (25.0)
Heart rate, beats/min	72.1 (12.4)	67.7 (13.0)	72.6 (12.6)	71.3 (12.9)	64.6 (12.6)	63.0 (10.9)
Serum cholesterol, mg/dL	240.3 (45.3)	261.0 (52.0)	235.5 (44.4)	201.7 (40.8)	205.4 (42.8)	165.2 (33.0)
CVD prevalence, %	11.3 (0.6)	19.4 (1.0)	7.7 (0.9)	5.4 (0.5)	4.9 (0.6)	0.6 (0.2)
ECG abnormalities, %	0.2 (0.03)	1.2 (0.3)	3.3 (0.6)	1.5 (0.3)	1.3 (0.3)	1.9 (0.4)
**60-year all-cause death rate, %**	99.3	99.8	99.8	99.8	99.8	95.7

ANOVA across countries for continuous variables: *p* for age = 0.0962 and *p* for all other variables <0.0001; chi-squared across countries for discrete variables: *p* for ECG = 0.024 and *p* for all other variables <0.0001.

**Table 2:** Major types of CVD death rates (per 1000) in 60 years and in 3 periods of 20 years each, in the 6 countries and the pool.

		CHD	STROKE	HDUE	STHD	Ratio CHD/STHD	CVD3
POOL	*N*						
Years 0–20	9063	95	38	20	57	1.65	153
Years 21–40	5963	182	100	81	180	1.01	363
Years 41–60	1261	95	101	155	256	0.37	381
**YEARS 0–60**	**9063**	**228**	**118**	**95**	**213**	**1.07**	**441**
**USA**							
Years 0–20	2571	128	24	27	51	2.48	179
Years 21–40	1693	217	74	86	160	1.36	377
Years 41–60	373	78	94	164	257	0.30	336
**YEARS 0–60**	**2571**	**282**	**86**	**108**	**194**	**1.45**	**476**
**FINLAND**							
Years 0–20	1677	173	36	16	52	3.33	225
Years 21–40	967	283	98	49	147	1.93	430
Years 41–60	169	154	83	166	249	0.62	402
**YEARS 0–60**	**1677**	**352**	**101**	**61**	**162**	**2.18**	**513**
THE NETHERLANDS							
Years 0–20	878	113	23	14	36	3.09	149
Years 21–40	592	218	73	59	132	1.65	350
Years 41–60	107	56	103	93	196	0.29	252
**YEARS 0–60**	**878**	**267**	**84**	**65**	**149**	**1.79**	**416**
**ITALY**							
Years 0–20	1712	57	64	27	73	0.78	130
Years 21–40	1111	143	110	103	212	0.67	356
Years 41–60	252	99	95	183	278	0.36	377
**YEARS 0–60**	**1712**	**164**	**131**	**120**	**252**	**0.65**	**416**
**GREECE**							
Years 0–20	1215	18	41	13	54	0.33	72
Years 21–40	939	120	125	115	240	0.50	360
Years 41–60	221	136	145	190	335	0.41	471
**YEARS 0–60**	**1215**	**136**	**164**	**137**	**300**	**0.45**	**436**
**JAPAN**							
Years 0–20	1010	23	75	12	87	0.26	110
Years 21–40	661	67	139	47	186	0.36	253
Years 41–60	139	30	81	67	148	0.20	178
**YEARS 0–60**	**1010**	**70**	**177**	**51**	**229**	**0.31**	**299**

CHD, coronary heart disease; HDUE, heart disease of uncertain etiology; STHD, STROKE + HDUE.; CVD3, CHD + STROKE + HDUE.; *N*, men alive at the beginning of each time period.

**Table 3:** Average age at death for the different types of CVD mortalities in 60 years.

	USA	Finland	The Netherlands	Italy	Greece	Japan	*p* of ANOVA across Countries
CHD	72.1	70.6	72.1	74.1	80.0	73.5	<0.0001
STROKE	77.5	75.9	76.7	75.7	78.7	74.8	0.0018
HDUE	79.1	80.7	79.6	79.9	83.6	78.8	0.0003
STHD	78.4	77.7	77.9	77.7	80.9	75.7	<0.0001
CVD3	74.7	72.9	74.2	76.3	80.6	75.2	<0.0001
***p* of ANOVA across 3 independent end-points**	<0.0001	<0.0001	<0.0001	<0.0001	<0.0001	0.0318	

CHD, coronary heart disease; HDUE, heart disease of uncertain etiology; STHD, STROKE + HDUE; CVD3, CHD + STROKE + HDUE.

**Table 4:** Cox models for CHD, STROKE and HDUE with all covariates, 2 reference and dummy variables identifying countries.

		CHD (*N* = 2066)				STROKE (*N* = 1068)				HDUE (*N* = 860)			
Variable	Delta	coeff	HR	lcl	Hcl	Coeff	HR	lcl	hcl	Coeff	HR	lcl	hcl
Age	5	0.0678	**1.40**	**1.34**	**1.47**	0.1167	**1.79**	**1.68**	**1.91**	0.1347	**1.96**	**1.83**	**2.11**
Never smoker	reference												
Ex smoker	1	0.0678	1.07	0.93	1.24	0.0726	1.08	0.89	1.30	0.0946	1.10	0.89	1.35
Current Smoker	1	0.4259	**1.53**	**1.36**	**1.72**	0.2071	**1.23**	**1.06**	**1.43**	0.3881	**1.47**	**1.25**	**1.74**
Vigorous physical activity	reference												
Moderate physical activity	1	0.1430	**1.15**	**1.01**	**1.31**	0.1096	1.12	0.96	1.30	0.1159	1.12	0.93	1.36
Sedentary physical activity	1	0.1928	**1.21**	**1.04**	**1.41**	0.1268	1.14	0.93	1.39	0.1857	1.20	0.96	1.51
BMI	3.5	0.0094	1.03	0.98	1.09	0.0002	1.01	0.94	1.09	0.0187	1.07	0.99	1.16
Systolic blood pressure	20	0.0121	**1.27**	**1.22**	**1.33**	0.0164	**1.39**	**1.31**	**1.47**	0.0122	**1.28**	**1.19**	**1.37**
Heart rate	13	0.0018	1.02	0.98	1.07	0.0062	**1.08**	**1.02**	**1.15**	−0.0034	0.96	0.89	1.03
Serum cholesterol	50	0.0046	**1.26**	**1.20**	**1.32**	0.0003	1.01	0.94	1.09	0.0011	1.05	0.97	1.14
CVD prevalence	1	0.5533	**1.74**	**1.53**	**1.98**	0.1825	1.20	0.95	1.51	0.4858	**1.63**	**1.28**	**2.06**
Silent ECG abnormality	1	0.3587	**1.43**	**1.06**	**1.93**	0.4049	1.50	0.99	2.28	0.5745	**1.78**	**1.12**	**2.81**
USA	1	−0.3575	**0.70**	**0.60**	**0.82**	−0.4358	**0.65**	**0.50**	**0.83**	0.2802	**1.32**	**1.00**	**1.75**
Finland	Reference												
Netherlands	1	−0.4223	**0.66**	**0.55**	**0.78**	0.4765	**0.62**	**0.46**	**0.83**	−0.1798	0.84	0.59	1.19
Italy	1	−0.5893	**0.55**	**0.47**	**0.65**	0.1019	1.11	0.88	1.39	0.5666	**1.76**	**1.35**	**2.30**
Greece	1	−1.0044	**0.37**	**0.30**	**0.44**	0.1084	1.11	0.89	1.40	0.3963	**1.49**	**1.14**	**1.94**
Japan	1	−1.1885	**0.30**	**0.23**	**0.40**	0.5160	**1.68**	**1.30**	**2.17**	−0.1909	0.83	0.57	1.20

Units of measurements as in Table 1. Delta for computation of hazard rates of continuous variables roughly corresponding to 1 standard deviation of variables (significant HR in bold).

**Table 5:** Cox model of non-CHD mortality with all covariates, 2 reference and dummy variables identifying countries.

		Non-CHD Deaths (N = 6926)			
Variable	Delta	coeff	HR	lcl	Hcl
Age	5	0.0995	**1.64**	**1.60**	**1.69**
Never smoker	reference	----	----	----	----
Ex smoker	1	0.1296	**1.14**	**1.05**	**1.23**
Current smoker	1	0.4258	**1.53**	**1.44**	**1.63**
Vigorous physical activity	reference	----	----	----	----
Moderate physical activity	1	0.0765	**1.08**	**1.01**	**1.15**
Sedentary physical activity	1	0.0884	**1.09**	**1.01**	**1.19**
BMI	3.5	−0.0140	**0.95**	**0.92**	**0.98**
Systolic blood pressure	20	0.0071	**1.15**	**1.12**	**1.18**
Heart rate	13	0.0052	**1.07**	**1.04**	**1.10**
Serum cholesterol	50	−0.0001	0.99	0.97	1.02
CVD prevalence	1	0.2448	**1.28**	**1.17**	**1.40**
Silent ECG abnormality	1	0.1881	**1.21**	**1.00**	**1.45**
USA	1	−0.1159	**0.89**	**0.81**	**0.98**
Finland	Reference	----	----	----	----
Netherlands	1	−0.1375	**0.87**	**0.78**	**0.97**
Italy	1	0.0866	**1.09**	**1.00**	**1.19**
Greece	1	−0.1389	**0.87**	**0.79**	**0.96**
Japan	1	0.2642	**1.30**	**1.17**	**1.45**

Units of measurements as in Table 1. Delta for computation of hazard rates of continuous variables roughly corresponding to 1 standard deviation of variables (significant HR in bold).

**Table 6:** Partial findings of discriminant analysis of CHD versus STHD, pooling all cohorts as a function of 11 risk factors (plus 2 references).

Variable	Removed Lambda	Removed F-Value	Removed F-Probability	Lambda Alone	F-Value Alone	F-Probability Alone
Age	0.9976	9.78	0.0018	0.9979	8.30	0.0040
Vigorous physical activity	reference					
Moderate physical activity	0.9999	0.27	0.6014	0.9999	0.01	0.9128
Sedentary physical activity	0.9981	7.63	0.0058	0.9959	16.52	0.0000
Never smoker	reference					
Ex smoker	0.9910	0.19	0.6669	0.9999	0.54	0.4635
Current Smoker	0.9972	11.26	0.0008	0.9966	13.69	0.0002
Body mass index	0.9999	0.21	0.6490	0.9981	7.72	0.0055
Systolic blood pressure	1.0000	0.00	0.9745	0.9997	1.29	0.2567
Heart rate	0.9999	0.41	0.5196	0.9975	10.21	0.0014
Serum cholesterol level	0.9587	171.55	0.0000	0.9486	216.34	0.0000
CVD prevalence	0.9964	14.34	0.0002	0.9923	31.00	0.0000
ECG silent abnormality	0.9999	0.20	0.6526	1.0000	0.00	0.9995

Units of measurement as of Table 1. Lambda is a test for evaluating the importance of risk factors in discrimination. Lambda with its F-value is a test describing the impact of risk factors on discrimination between the two end-points; removed lambda with its F-value and F-probabilities when the variable is removed, lambda alone with its F-value and F-probabilities when only that variable was used; low probabilities (for example <0.05) correspond to the high importance of the variable.

**Table 7:** Average baseline serum cholesterol level (mg/dL) of men who died from the 3 CVD end-points.

	All Countries	USA	FIN	NED	ITA	GRE	JAP	*p* of ANOVA across Countries
**CHD**	243.3 (54.3)	247.9 (44.6)	271.3 (53.0)	242.9 (46.5)	209.6 (42.2)	213.6 (53.1)	167.7 (34.0)	<0.0001
**STROKE**	216.3 (50.0)	242.0 (43.6)	259.3 (45.0)	233.2 (36.6)	204.5 (38.8)	204.4 (38.5)	165.3 (31.3)	<0.0001
**HDUE**	221.6 (52.5)	240.5 (46.0)	261.4 (58.4)	242.5 (55.6)	198.7 (38.8)	204.3 (43.0)	165.9 (29.9)	<0.0001
**P of ANOVA** **across** **3 end-points**	<0.0001	0.0412	0.0133	0.2774	0.0134	0.0904	0.8586	

Standard deviation in brackets (SD). USA, United States; FIN, Finland, NED, The Netherlands; ITA, Italy; GRE, Greece; JAP, Japan.

## Data Availability

Internal rules of the research group do not allow to share original data with third parties.
